# Investing for population mental health in low and middle income countries—where and why?

**DOI:** 10.1186/s13033-022-00547-6

**Published:** 2022-08-11

**Authors:** Melvyn Freeman

**Affiliations:** University of Stellenbosch, Private Bag X1, Matieland, Stellenbosch, 7602 South Africa

**Keywords:** Mental health financing, Social determinants, Low and middle income countries, Promotion, Serious mental disorder, Common mental disorder

## Abstract

**Background:**

Policy makers intent on improving population mental health are required to make fundamental decisions on where to invest resources to achieve optimal outcomes. While research on the effectiveness and efficiency of interventions is critical to such choices, including clinical outcomes and return on investment, in the “real world” of policy making other concerns invariably also play a role. Politics, history, community awareness and demands for care, understanding of etiology, severity of condition and local circumstances are all critical. Policy makers should not merely rely on previous allocations, but need to take active decisions regarding the proportion of resources that should be allocated to particular interventions to achieve optimum outcomes. Given that scientific evidence is only one of the reasons informing such decisions, it is necessary to have clear and informed reasons for allocations and for making cases for new mental health investments.

**Main body:**

Investment allocations are unlikely to ever be an exact science. Alternatives therefore need to be rationally weighed up and reasoned decisions made based on this. Using prevalence data and the distribution of mental health resources in South Africa as a backdrop and proxy, investment proposals are made for LMICs with due consideration given to inter alia the social determinants of mental health, the needs and potential benefits of investments in people with severe verses common mental disorder, mental health promotion and disease prevention and to other areas that may impact on population mental health, such as management.

**Conclusion:**

Based on a range of arguments, it is proposed that mental health investments should follow the following approach. A mental health-in-all-policies method must be adopted. There should be no more than a 20% gap in the humane and human rights oriented care, treatment and rehabilitation of people with severe mental disorder. A minimum additional amount of 10% of the amount spent on severe mental disorder should be allocated to treating people with common mental disorder. Screening for mental disabilities should take place within all chronic care services. A minimum of 3% of the budget spent on severe mental disorder should be spent on promotion and prevention programmes. An additional 1% of the allocation for severe mental disorder should be provided for managing/driving the mental health programme.

## Background

Policy makers and advisors seeking to improve population mental health status are faced with difficult choices regarding where and how to invest (scarce) resources that bring optimal health as well as economic and social benefits—while also being locally appropriate, affordable and feasible. Basing investment decisions on scientific evidence is laudable and certainly to be aimed towards, however despite progress being made on returns on investment through for example the WHO OneHealth Tool [1], and detailed costing that has been done on the financial resources that would be required to close the mental health treatment gap in some countries [2], in the “real world” of policy making there are invariably a range of considerations that fall outside of scientific certainly and hence some form of “rational subjectivity” is both inevitable and necessary. The argument put forward here is based mainly on the author’s 15 years of experience heading the mental health unit, and later non-communicable diseases section (including mental health), at the National Department of Health in South Africa.

At the same time as advocating for more resources for mental health, which is fundamental given current low allocations, it is essential to recognize that funding a full package of required mental health interventions is a long way off for most countries. A phased approach, with a clear determination of where and how best to utilize resources, is thus necessary. While the high burden of disease attributable to mental health is primarily due to high prevalence conditions such as depression and anxiety, in most LMICs countries by far the majority of expenditure is on treatment/care of severe conditions such as schizophrenia and bi-polar mood disorder. Budgets for prevention and promotion are usually minimal or even non-existent in most LMICs.

This article addresses the issue of how and where best to allocate mental health budgets and which areas to advocate for new resources, should the opportunity arise. Rather than merely repeating apportionments to current allocations in mental health, as most countries tend to do year after year, it is incumbent on policy makers to assess where and how existing resources should be optimally allocated and if required, to shift resources accordingly.

In broad terms, decisions to achieve population mental health must be made with respect to allocations to (at least) (1) The social determinants of mental health (as these may potentially assist in improving mental health status); (2) Severe mental disorders; (3) Common mental disorders; (4) Prevention and Promotion and (5) Management. Within each of these categories there are numerous options and decisions that need to be made with respect to the specifics of interventions, but how best to initially allocate within these broad categories is fundamental. The approach adopted is that where research is available this is utilized, but there are also numerous other considerations in policy making, and these too are critically important.

Using the prevalence of mental disorder and distribution of mental health resources in South Africa as a backdrop and proxy, this article presents broad investment proposals for LMICs. These proposals are based on analysis of a wide range of factors, including needs, cost effectiveness and whether investing into the social determinants of mental health may be a preferable investment option to more or improved mental health services. While these proposals may be relevant to many LMICs, it is strongly acknowledged that each country has different needs and circumstances. They are thus not presented as fixed formulae to be utilized blindly by LMICs, but as broad submissions requiring local reflection, input and interrogation.

### Prevalence of mental disorder and expenditure on mental health in South Africa

The 12 months prevalence of mental disorder in South Africa has been estimated at 16.5% [3]. Importantly for this paper, around 2% of the population were assessed to have severe mental disorder, while the remainder had a range of conditions frequently referred to as common mental disorders including depression, anxiety and substance abuse disorders. While no national data is available, the 12-month prevalence of child and adolescent mental disorders in the Western Cape is reported to be 17% [4].

South Africa allocates around 5% of its health budget to mental health—this being USD615.3 million in the 2016/17 financial year [5]. This amounts to USD13.3 per capita of the uninsured population. Inpatient care accounts for 86% of mental healthcare expenditure, with nearly half of total mental health spending occurring at the psychiatric hospital-level. The vast majority of admissions are for severe mental health conditions. Only around 8% of the mental health budget is spent within primary health care [5]. This includes down referrals from hospitals for people with severe mental disorders into primary care in clinics and community health centres and includes costs of medicines. As with hospital care (in psychiatric and general hospitals where psychiatric care is provided), the vast majority of primary care mental health expenditure is spent on treatment for people with severe mental disorder. Hence despite the high prevalence of common mental health conditions, expenditure on people with these conditions is minimal. Moreover, it is evident that given the very high of percentage of the health budget spent on in-patient care, very little expenditure is on prevention and promotion.

### Funding for improved mental health

It is widely recognized that mental health is neglected, under-resourced and underfunded, particularly in low and middle income countries (LMICs) [6, 7]. The treatment gap—that is the absolute difference between the true prevalence of a disorder and the treated proportion of individuals affected by the disorder [8]—exceeds 50% in all countries, approaching 90% in the least resourced countries [9, 10]. Over a number of studies the median untreated rate for schizophrenia and other non-affective psychosis has been found to be 32%, major depression 56%, dysthymia 56%, bipolar disorder 50%, GAD 58% and OCD 60%, alcohol abuse and dependence 78% [2].

The South African Health and Stress Study (SASH) found a treatment gap of 75% for common mental disorders [11]. However, a more recent analysis suggests that the gap for mental disorders, epilepsy and intellectual disability is close to 92% [5]. The vast majority of untreated disorders are persons with common mental disorder.

Given that country budgets are finite and in demand from many competing sectors, including from areas that affect mental health such as poverty alleviation, job creation, housing, education, social security, safety, nutrition [12], making a case for investment in mental health requires careful deliberation and weighing up of evidence from a wide range of perspectives. Allocating more resources to treatment at the expense of social determinants or vice versa might widen rather than narrow the treatment gap.

Closing the gap through more efficient and effective (including cost effective) approaches, as well as by eliminating wasteful expenditure, is necessary and possible. For example, resources can be spread through decentralization using task shifting or task sharing approaches [13, 14] or through more competitive tendering for purchasing of medication or equipment [15, 16]. The treatment gap can also be narrowed through Universal Health coverage initiatives such as National Health Insurance that allows more equitable distribution of human and financial resources and hence wider service coverage [17]. As essential as these innovations are though, and as much as the treatment gap also arises as a result of stigma [18], and cultural factors [19] that prevent people seeking treatment, for most LMICs additional resources are required to adequately redress unmet need. Two key question then arise. Where should current resources that are allocated to mental health be spent to achieve optimal outcomes? And, to which areas should additional resources be allocated should these be come available for mental health,?

Given global fiscal constraints and an area of health that has relatively low mortality, is not infectious and has high stigma, it is critical that resource allocation—if not to be based on pure science alone and is not merely the whim of a donor or politician—must be carefully thought through and debated.

Ideally, fiscal decisions by governments should be based on comparative analyses and evidence in which a wide array of investment proposals across different sectors are weighed against each other. In such circumstances, the health benefits would be included in the cases made by other departments as part of a health-in-all-policies approach [20] while health would calculate the proven benefits to other sectors such as education or the economy in its bids. Any specific proposal for additional resources would thus not be evaluated against itself, that is either funded or not based on the merits of the specific proposal, including its cost benefit, but scrutinized and compared against every other case both across sectors and within a sector. Criteria would be objectively weighted and summed to allow comparisons, and rational decisions would be based on a complete picture.

This ideal however, is unlikely to be realized soon (or perhaps ever), as allocating fair weighting will inevitably remain highly subjective, but also because in addition to scientific evidence, decisions about prioritization invariably also include electoral promises, party political directions, media pressures, personal preferences and priorities (especially of people in influential positions), expediency, strength of advocacy groups and commitment to fulfilling targets of internationally agreed resolutions, conventions or goals. And as much as scientists or researchers may wish otherwise, weighting such variables objectively will in all likelihood never be possible. In addition, proposed budget shifts are normally moderated by policy makers typically reluctant to alter the funding of line items from previous allocations in order to maintain fiscal and other stability and discipline and to disrupt existing services as little as possible.

As far as this author is aware, despite good evidence showing critical impacts of economic and social determinants on mental health [11], there are as yet no studies that show the relative benefit to population mental health of investments in these determinants vis-à-vis those into direct mental health care and/or prevention interventions. Given the critical importance to countries in making reasoned investment decisions in this regard, and that a comprehensive trial would be highly complex and perhaps not possible at all, a rapid and rough approach is to calculate the potential impacts of added investments into the social determinants of mental health against similar investment in mental health programmes directly. So for example, if a substantial but potentially realizable 20% of the current mental health budget in South Africa was to be invested in various targeted social determinants, with the specific objective of improving mental health, what would the likely impact be on mental health?

The full mental health budget in South Africa accounts for around 0.003% of the total country budget. Twenty percent would thus be 0.0006% of total expenditure. Key social determinants that impact on mental health such as poverty alleviation, job creation, housing, education, social security, nutrition, safety, justice and so on already constitute by far the majority of government expenditure [21] . Consequently, the addition of a small fraction of one percent to the broad social determinants would be such a miniscule addition to these services that it is difficult to imagine how such addition would change the social determinants at all, and certainly not to the degree that they would in turn knock on to positively impact on population mental health. Should an amount equivalent to an extra 20% of the mental health budget become available it would thus appear prudent to carefully invest this in mental health programmes themselves—whether this be more direct prevention/promotion or to treatment and care programmes. This is not meant to weaken in any way the importance of the impacts of social determinants on mental health, but only that the magnitude of a potential investment for mental health through the conduit of social determinants may not significantly change population mental health. Moreover, in terms of the vicious cycle that sees people with poor mental health being unable to overcome obstacles to social and community cohesion and to being less able to contribute to social and economic growth and development, direct prevention programmes or treatment may be critical interventions in ending a social and economic down-spiral, albeit from the opposite pole from the social determinants. In addition, every person has the right to the Highest Attainable Standard of Health and this includes a right to mental health care, treatment and rehabilitation [22].

New funding in health usually results from an unanticipated crisis situation or a new or exponentially growing health problem combined with evidence of cost effectiveness or social benefit and security. For example in South Africa, following investment cases being made to the Treasury, significant additional resources were made available for the roll-out of anti-retroviral therapy to control HIV and for pilot sites in preparation for National Health Insurance [23] . Almost every country in the world has had to allocate or find additional resources to deal with the COVID 19 pandemic crisis that has hit the globe. These additional allocations have been necessary to avert a global catastrophe of monumental social and economic proportions.

In terms of mental health, the World Bank and the World Health Organization have provided wide-ranging arguments for why mental health now constitutes a global crisis [4]. While clearly not of the same proportions as COVID 19, they make the case for more investment in mental health services based on its high prevalence; large burden, especially in terms of years lived with disability; availability of effective treatments; high costs of non-treatment; proven prevention programmes and very importantly the cost benefit of mental health interventions for certain conditions [4].

There is no internationally agreed formula for determining the proportion of a total budget that should be allocated to health [24] or the appropriate proportion of the health budget for mental health, or within mental health between different areas. Countries need guidance and various attempts have been made by economists and organizations to try to draw fair and reasonable resources to mental health. These attempts are extremely important but have their limitations in terms of where and how to invest mental health resources.

### Budget allocations for mental health

A commonly proposed approach with respect to the proportion of the health budget that should be spent on mental health has been to suggest that the health budget should mirror the percentage of DALYs contributed by mental and neurological disorders (7.4%–14%) [25, 26] . Vigo et al have made the compelling argument that in fact actual DALYs are at the higher end of this range (13%), level with cardiovascular and circulatory diseases,[27] and therefore current budget allocations in all countries are far too low. But even at the lower level, the proposal to fund proportionate to the burden of disease would require huge increases in mental health service allocations from the current average of 0.5% in low-income countries and even from the 5.1% in high income countries; and indeed in South Africa from the 5% it currently spends [28] . Though attractive from a mental health perspective, the argument to fund disease/disorder categories relative to their burden is simplistic and reductionist and is unlikely to shift real allocations [29] . For example, infectious diseases *do* warrant special prioritization—not least because DALY calculations do not include people who would become infected should comprehensive control not be introduced. Investing in HIV prevention and treatment in South Africa or the global COVID 19 virus serve as good examples of the importance of investments into communicable diseases in order to avoid a global health and security crisis, as well as to avoid much higher DALYs for these conditions in the future.

Moreover, the approach of funding based on burden does not take into account that the price of treatments and intensity of care needed for different diseases differs substantially. For example, the actual costs of treating non-communicable diseases such as cancer or severe kidney disease through chemotherapy and dialysis, relative to the costs of a lay counsellor treating mild or moderate depression, need to be carefully considered when allocating resources. Budgeting using DALYs alone would in all likelihood result in conditions that are cheap and relatively easy to treat receiving more resources than complex health conditions that require significant resources. This in turn is likely to result in higher mortality rates for conditions that would of necessity have had their budgets cut to accommodate a burden-based budgeting approach. This would alter (and increase) future DALY calculations for such conditions.

The response needed is in fact much more complex than a simple allocation based on burden alone. The reasons that higher income countries allocate a higher percentage of their health budgets to mental health than LMICs is important [30], but there are reasons for this. Most LMIC are already battling to fund treatment for even high mortality conditions. Using proportionate burden-based allocations suggests that there should be a transfer of resources from certain health conditions to others. This could be highly detrimental to people in LMICs where most areas of health are underfunded. What is in fact required to increase the proportion of the health budget that goes to mental health is not a reallocation of health resources, but additional funding being allocated to health that is dedicated to mental health. But this too is complex as countries have fiscal constraints that don’t easily accommodate additional funding to health, and if this does occur this can also negatively impact the social determinants of mental health.

Even within mental health, the logic of budget allocation based on DALYs would result in major incongruities and radical and potentially irrational reversals from current global practices. For example this approach would result in a relatively small proportion of the mental health budget being allocated for the care and treatment of schizophrenia as it constitutes only 7.4% of mental health DALYs, relative to say depressive disorders that constitutes 40.5% [21]. If this was the basis for budgeting this allocation would occur despite a person with treatment-resistant schizophrenia possibly requiring multiple and long stay hospitalizations, the intervention of a psychiatrist, lifelong medication, and psychosocial support in the community; whereas a person with depression might only require counseling from a lay community counselor over a period of a few weeks. The cost of an average case for treatment of depression in South Africa using the WHO GAP Action programme treatment components is around $87 compared with $396 for psychosis [31]. But it would be illogical to prioritize and treat a person with depression over a person with schizophrenia simply because it is cheaper and brings better investment returns (see later) without taking many other considerations into account.

Another budgeting approach has been to illustrate the cost benefit of mental health interventions. Results of economic modeling show that for every unit spent on treatment of depression the cost-benefit ratios amount to 2·3–3·0 to 1 when economic benefits only are considered, and 3·3–5·7 to 1 when the value of health returns is also included, with the highest rewards being in lower-middle income countries [32]. For severe mental health conditions the returns are not nearly as high, and in fact there may not be positive financial returns at all on such investment [33]. A return on investment based approach to budgetting could result in a county’s limited mental health resource being all (or almost all) being utilized for treating common mental health conditions on the grounds that the economic return is greatest. But cost benefit or return on investment can and must be only one of many criteria for deciding where and how to intervene in mental health. For instance, the disruption that a person with untreated schizophrenia might cause in a community, their higher risk of other physical morbidities, higher mortality and the fact that without treatment they might be abused or imprisoned also have to be considered. While the above cost benefit argument implies that investment in treatment of depression and anxiety will not cost the country anything in the longer term due to the social cost benefit, and possibly that this investment should be in addition to investments in severe mental disorder rather than instead of such investment, an immediate upfront budget is nonetheless required from the State, given that most LMIC countries currently invest proportionately very little in common mental disorder [34].

The expected resource needs of a package of care based on target levels of coverage has been calculated for certain countries [35] . A defined package of cost-effective interventions for schizophrenia, depression, epilepsy and alcohol use disorders in Sub-Saharan Africa and South Asia was found to be around $3-4 per person. In upper-middle income countries, such a package is expected to be at least double this amount [20]. Using a mental health systems planning tool (OneHealth), a recent study on costs of mental health at target levels in 6 LMICs ranged from 0.14 to US$5 per capita with a yield of between 291 and 947 healthy years per million population gained [36]. The actual cost makes this an aspirational rather than a realistic target, even though the authors suggest that this may be phased in. Moreover, this approach has not included costs for prevention and potential cost savings that should accrue from this.

### Prevention and promotion in mental health

Promoting mental health and preventing mental illness is a burgeoning field that warrants inclusion in the mental health programme of every country. The World Health Organization have documented a number of effective programmes [37] while the emerging evidence from low resource settings has been well summarized and documented by Petersen et al [38]. The importance of prevention in mental health has also been emphasized in the Lancet Commission on Mental Health and Sustainable Development [12]. Moreover, the internet and mobile based interventions are showing promise for highly accessible interventions for prevention of depression for example [39] that may also be feasible in LMIC as global internet use increases. A review of preventive/promotive interventions provided at the population- and community-levels in LMICs concluded that such interventions have an important role to play in promoting mental health, preventing the onset, and protecting those with Mental Neurological and Substance abuse disorders [40]. However, the need for further research on interventions in LMICs is highlighted.

### Proposal for investment into areas of mental health

In the absence of a scientifically workable formula for mental health, allocations due to the nature and varying influences on such decisions, broad proposals for public investment in LMICs are recommended below. These suggestions utilize assessments of previous proposals including those based in clinical and cost effectiveness research, a commitment to the most vulnerable members of society, current practice in most LMIC and makes a judgment on what may be feasible and acceptable in LMICs going forward. It keeps the amounts requested at modest levels, as in a climate of low growth and the COVID 19 pandemic not only will additional resources be difficult to obtain due to competing demands, but when the mental health budget increases some other budget line item must decrease, which could in turn impact negatively. Even within health, given high comorbidities between mental and physical health [7], reducing physical health expenditures in almost any disease area will negatively impact mental health.

Thorough research may in time show that investing in social determinants outweigh direct mental health investments, but until then it is proposed that should funds become available to improve mental health, direct investments should be made into mental health programmes. Notwithstanding, a health in all policies approach is still emphasized as deterioration in the social and economic determinants will invariably lead to a decline in mental health status.

The starting point of this proposal is that there should not be more than a 20% gap in the humane and human rights-oriented care, treatment and rehabilitation of people with severe mental order. As reported above, the current global median untreated rate for schizophrenia and other non-affective psychosis has been found to be 32%, while for bipolar disorder this gap is 50%. Given the serious implications that such conditions have on the individual, their family and community, no person with a severe mental disorder should be left untreated. However, given that the majority of the mental health budgets in LMICs are already expended on these conditions, and that some people may not want, or refuse, treatment, it is proposed that this target be 80%. Given a history of poor-quality care and abuse of human rights in many LMICs [41], this intervention, including its costing, must comply with internationally acceptable standards and norms. Moreover, the majority of the budget should be spent in caring for people in the community rather than in psychiatric hospitals [42]. (See Table 1 for further explanation).

**Table 1:** Proposed allocations to mental health and the rationale

Proposal	Explanation
i) A mental health-in-all-policies approach must be adopted	• Social determinants of mental health are very important to achieving population mental health and hence policies adopted in sectors outside of health must consider their impacts on mental health. For example education, housing and labour can adjust their policies and programmes in ways that improve mental health outcomes without significantly adding to their output costs. Mental health must be included in structures currently existing or that are being set up in countries to deal with the social determinants of non-communicable diseases, such as National Health Commissions [43] or Health Promotion Foundations [44]
ii) There should be no more than a 20% gap in the humane and human rights oriented care, treatment and rehabilitation of people with severe mental order	• Ideally every person with severe mental disorder should have access to comprehensive mental health care, treatment and rehabilitation. However, given current large treatment gaps in LMICs [45] there should be a minimum initial target of 80% • Quality of care and the human rights of users should be determined at internationally acceptable norms and standards • This target should not include people who experience psychotic symptoms that are part of cultural expression or are non-psychotic hallucinations or delusions • The vast majority of users, if not all, should live in communities • Users must be treated for both their physical and mental health care needs • Specialist personnel such as psychiatrists and psychologists should be available to support less specialized personnel through task sharing and task shifting while also taking referrals of more complex cases
iii) A minimum additional amount of 10% of the amount spent on severe mental disorder should be allocated to treating people with common mental disorder	• This percentage appears highly asymmetrical and unfair especially as this group would usually constitute 5–10 times more in actual numbers than people with severe mental order and usually includes highly vulnerable people such as victims of violence. However, this proposal is based on the cost of care itself (i.e. significantly higher for severe mental disorder); on the implications of not treating a person with severe mental disorder; and on the fact that for treatment of common mental disorder most LMIC countries will be starting from a very low base • Cost benefit will be high • These services need to be built up and further resourced over time • Task shifting and task sharing must form an important part of the care and treatment for people with common mental illness
iv) Given the high co morbidity between mental and physical health and the reasons for this [46], screening for mental disabilities should take place within all chronic care services	• Screening for mental health should be included in services for both communicable diseases such as HIV and TB and Non-communicable diseases such as hypertension and diabetes. Treatment should then be offered/provided to those that are screened positive [47]. This would allow for a rational and logical process to increase the numbers receiving care especially for common mental disorder
v) A minimum of 3% of the budget spent on severe mental disorder should be spent on promotion and prevention programmes	• As with common mental disorder, it is expected that the economic return on investment through prevention and promotion will be far higher than expenditure on severe mental disorder, and indeed more desirable [48], however this proposal acknowledges that mental health resources will in all likelihood be in short supply and taking away from treatment of severe mental disorder, even if it is for prevention, will have medical, social, economic and even political implications • Prevention and promotion will also be starting from a very low base in most LMIC and hence even 3% of the treatment amount may initially be difficult to absorb into effective prevention programmes. While financial returns on such investment is often difficult to measure, available research does indicates good value for money [49, 50] while also approaching mental health from the most humane way possible [38] • Stigma reduction programmes must form part of this resource
vi) An additional 1% of the allocation for serious mental disorder should be provided for driving the mental health programme	• This allocation will for resourcing leadership, stewardship and assistance from policy development through to programme implementation as well as monitoring and evaluation. Without this, mental health interventions will fail

Based on this spend (and this will vary from country to country), an *additional* initial amount of 10%, calculated from the amount spent on severe mental disorder, should be allocated for the needs of persons with common mental disorder. This appears highly asymmetrical and unfair especially as this group would usually constitute 5-10 times more in actual numbers than people with serious mental order and usually includes highly vulnerable people such as victims of violence and abuse, but this proposal is based firstly on the cost of care itself (ie significantly higher for serious mental disorder), on the implications of not treating a person with serious mental disorder, and that for treatment of common mental disorder most LMIC countries will be starting from a very low base – for some countries nothing at all. Hence in most countries, including South Africa, 10% of the expenditure on severe mental disorder will be a huge initial step forward in the treatment of common mental health conditions. (See Table 1 for further explanation). This proposal is made with the full understanding that the cost benefit will not be nearly as great for severe mental disorder as it would for common mental disorder.

It is proposed that an additional amount equal to 3% of the expenditure on severe mental disorder should be allocated for mental health promotion and illness prevention programmes. As with common mental disorder it is expected that the economic return on investment will be far higher than expenditure on severe mental disorder, and indeed prevention is more desirable, however this proposal acknowledges that mental health resources will in all likelihood be in short supply and taking away from treatment of severe mental disorder, even if it is for prevention, will have medical, social, economic and even political implications. Prevention and promotion will also be starting from a very low base in most LMIC and hence even 3% of the treatment amount may initially be difficult to absorb into effective prevention programmes (See table 1 for further explanation).

Finally, this proposal recommends that an additional 1% of the allocation for severe mental disorder be provided for driving the mental health programme. Without leadership and intensive stewardship from levels of national policy through to district implementation, and based on accurate information, effective mental health interventions will flounder.

## Conclusion

Recommendations for the allocation to different areas of mental health and the rationale for them have been made. It has been argued that “subjective rationality” is essential to “real world” health decision making. Research results are critical, but policy making also involves political and social considerations as well as practical experience.

For most LMICs the first step towards the achievement of these proposals will be to establish how much they are spending on mental health; where, on what disorders and how much goes on prevention and promotion. This will not be easy as mental health budgets are seldom ring-fenced and countries provide mental health care services at different levels and places within the health system. Expenditure will thus need to be calculated from the sum of sources including psychiatric hospitals; people admitted to general hospitals with mental health conditions; forensic mental health services; acute cases within primary care; chronic cases within primary care; (direct) promotion programmes in health or any other departments; (direct) prevention programmes in health and so forth. Such analysis recently done in South Africa provides a critical beginning to reallocating current budgets based on policy and prevalence and for deciding on which area(s) new investment case(s) must be made.

## Data Availability

All data used is from secondary materials and is available from references.
